# Assessment of Geochemical Limitations to Utilizing CO_2_ as a Cushion Gas in Compressed Energy Storage Systems

**DOI:** 10.1089/ees.2020.0345

**Published:** 2021-03-17

**Authors:** Chidera O. Iloejesi, Lauren E. Beckingham

**Affiliations:** Department of Civil Engineering, Auburn University, Auburn, Alabama, USA.

**Keywords:** CO_2_ sequestration, CO_2_ utilization, energy storage, Geochemistry, reactive transport

## Abstract

Compressed energy storage (CES) of air, CO_2_, or H_2_ in porous formations is a promising means of energy storage to abate the intermittency of renewable energy production. During operation, gas is injected during times of excess energy production and extracted during excess demands to drive turbines. Storage in saline aquifers using CO_2_ as a cushion or working gas has numerous advantages over typical air storage in caverns. However, interactions between CO_2_ and saline aquifers may result in potential operational limitations and have not been considered. This work utilizes reactive transport simulations to evaluate the geochemical reactions that occur during injection and extraction operational cycles for CES in a porous formation using CO_2_ as a cushion gas. Simulation results are compared with similar simulations considering an injection-only flow regime of geologic CO_2_ storage. Once injected, CO_2_ creates conditions favorable for dissolution of carbonate and aluminosilicate minerals. However, the dissolution extent is limited in the cyclic flow regime where significantly smaller dissolution occurs after the first cycle such that CO_2_ is a viable choice of cushion gas. In the injection-only flow regime, larger extents of dissolution occur as the fluid continues to be undersaturated with respect to formation minerals throughout the study period and porosity increased uniformly from 24.84% to 33.6% throughout the simulation domain. For the cyclic flow conditions, porosity increases nonuniformly to 31.1% and 25.8% closest and furthest from the injection well, respectively.

## Introduction

Compressed Energy Storage (CES) in subsurface formations is a promising means of long term, large capacity energy storage required to increase reliance on renewable energy and eliminate the fluctuation associated with renewable energy production (Schoenung and Hassenzahl, 2001; van der Linden, 2006; Cavallo, 2007; Succar and Williams, 2008). Potential geological storage formations include caverns and porous formations, such as depleted gas reservoirs and saline aquifers (Bary *et al.*, 2002; Ozarslan, 2012; Pfeiffer and Bauer, 2015; Wang and Bauer, 2017). Porous saline aquifers are particularly favorable due to their large potential storage capacity and the ubiquity of potential storage sites (Succar and Williams, 2008; Wang and Bauer, 2017; Mouli-Castillo *et al.*, 2019; Sopher *et al.*, 2019). Porous saline aquifers, however, have not been previously used for CES and involve additional complexities compared to storage in caverns, including multiphase flow and geochemical reactions that are not well understood and may impact system operation or efficiency (Allen, 1981; Beckingham and Winningham, 2020).

**FIG. 1.**
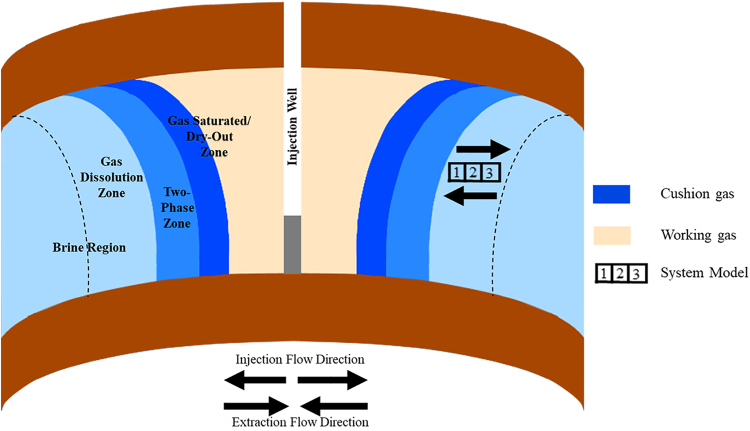
Schematic of idealized anticline saline aquifer compressed energy storage system showing the delineation of the working gas, cushion gas, and brine regions. Also shown is the conceptualized location of the simulated reactive transport model grid location. The *dotted line* illustrates the extent of gas dissolution into the brine.

CES systems store and produce energy through injection and extraction of a gas, referred to as a working gas. When energy production exceeds demands, the gas is injected into the storage formation and then extracted and used to drive a turbine and recover energy when demands exceed production. To establish the storage system, a cushion gas that will remain in the formation throughout the system operation is first injected followed by the working gas. The cushion gas may be the same or different in composition as the working gas but mainly serves to ensure adequate operational pressure to facilitate extraction (Carden and Paterson, 1979). During injection of the cushion gas into the brine saturated porous aquifer, three distinct zones are created as the injected gas pushes brine away from the injection well (Cui *et al.*, 2018). This includes a gas saturated or “dry-out” zone near the well surrounded by a two-phase gas and brine mixing zone and single-phase brine saturated zone furthest from the well (Fig. 1). The working gas, the same or different in composition to the cushion gas, is then injected into the porous aquifer and recycled for energy generation. Previous studies have identified that a third of volume of the injected gas is stored in the porous saline aquifer as cushion gas to ensure isobaric extraction during operation (Carden and Paterson, 1979). At the gas/brine interface, gas dissolves into the brine phase and water into the gas phase, controlled by their mutual solubilities. Depending on the choice of working or cushion gas and storage formation, the properties of the gas phase may vary from ideality and some phases may even exist as supercritical phases in the storage formation where air, CO_2_, H_2_, and gas mixtures have been considered as working or cushion gases (Beckingham and Winningham, 2020).

CO_2_ is a promising choice of cushion gas where previous studies on utilization of CO_2_ as a cushion gas have shown that its properties may increase operational efficiency (Laille *et al.*, 1988; Dussaud, 1989; Oldenburg *et al.*, 2004). At depths of typical storage formations, CO_2_ will exist as a supercritical fluid, with a high density and high compressibility that translate to large storage capacity (Suekane *et al.*, 2005; Oldenburg and Pan, 2013). Compressibility is an important property to consider in selecting the gas utilized in CES systems to minimize pressure variability during injection and extraction cycles, particularly for the selection of a cushion gas (Oldenburg, 2003), where highly compressible phases will maintain pressures and enhance operational efficiency. The high heat capacity of CO_2_ (He *et al.*, 2018) is also anticipated to favorably impact operational efficiency in comparison to the utilization of other working gases. Utilization of CO_2_ as a cushion gas would provide additional environmental benefits through the reduction in anthropogenic greenhouse gas emissions and economic advantages in the form of the cost of recovering the cushion gas upon the end of project life span as the injected CO_2_ can be permeability sequestered in the formation. This is in addition to additional benefits from carbon tax credits, as the cushion gas is injected periodically to sustain operational pressure during the operational life span (Metcalf, 2009).

Injection of CO_2_ in saline aquifers has been studied extensively in the context of geologic CO_2_ sequestration. These investigations have revealed dissolution of CO_2_ into formation brine following injection that lowers pH and results in the dissolution of carbonate and aluminosilicate minerals, buffering pH, and creating conditions favorable for precipitation of secondary minerals (Ketzer *et al.*, 2009; Ellis *et al.*, 2011; Farquhar *et al.*, 2013; Gharbi *et al.*, 2013; Liu *et al.*, 2013; Xiong *et al.*, 2018; Zou *et al.*, 2018; Fazeli *et al.*, 2019). These geochemical reactions may result in modifications to pore structures and connectivity (Luquot and Gouze, 2009; Gharbi *et al.*, 2013; Nogues *et al.*, 2013; Xiong *et al.*, 2018) that alter permeability (Ketzer *et al.*, 2009; Liu *et al.*, 2013; Zou *et al.*, 2018) and widen fractures (Ellis *et al.*, 2011; Deng *et al.*, 2018; Fazeli *et al.*, 2019) in subsurface systems. In energy storage systems, these reactions may intensify the migration of the cushion gas away from the injection well and further into the formation or enhance trapping of the cushion gas near the well, depending on if reactions result in increases or decreases in formation permeability^11^. If the migration of the cushion gas into the formation is promoted, the gas remaining near the well in the desired cushion gas zone that is required to maintain the pressure necessary for efficient cycling of the working gas during operation will decrease, reducing operational efficiency. This would also require more frequent injections of additional cushion gas to establish the pressure plume for operation. If migration of the cushion gas into the formation is further inhibited, operational efficiencies may actually increase as the pressure will be more easily maintained and the need to inject additional cushion gas will be reduced.

CO_2_-cushioned CES systems can be carried out in the same porous aquifers as CO_2_ sequestration (Kabuth *et al.*, 2017) but the resulting geochemical conditions, reactions, and impact of resulting reactions are unknown. As the CES system is established, the injection of the cushion gas to develop the gas bubble mimics that of geological CO_2_ sequestration systems with unidirectional flow. Following this, however, CES systems deviate with injection of a potentially different composition working gas that is cycled over periods of hours to months for energy storage or production (Carden and Paterson, 1979; Crotogino *et al.*, 2010; Carr *et al.*, 2014). The rate, extent, and impact of potential geochemical reactions at the gas dissolution zone under these flow conditions have not been considered. In this work, the geochemical influence of the cyclic flow regime of CES systems on geochemical reactions at the cushion gas–brine interface in the porous aquifer is considered and compared to reactions for a CO_2_ sequestration system. This region is selected as it is anticipated to be the region most impacted by mineral dissolution reactions. A reactive transport simulation for the cyclic flow conditions corresponding to energy storage in porous formations is developed and used to examine the evolution of formation brine, mineral volume fractions, and mineral porosity. Simulation results are compared quantitatively and qualitatively to those for a similar system considering geological CO_2_ sequestration in the same formation to deduce differences or similarities in potential geochemical reactions due to the flow regimes of the two systems.

## Materials and Methods

### Sample

The sample considered in this study is from the Paluxy formation, a prospective CO_2_ storage reservoir at the Kemper Power Plant in Mississippi (Project ECO_2_S). The formation is stratigraphically located between the Washita-Fredericksburg and Mooringsport formation in the Mississippi Gulf Coast (John Warner, 1993). This sample was the subject of previous work in Qin and Beckingham that utilized imaging to characterize sample properties (Qin and Beckingham, 2019) and simulated the rate and extent of geochemical reactions in the storage reservoir following CO_2_ injection (Qin and Beckingham, 2021). The sample was extracted from a depth of 5048 ft from well MPC 10-4 and is composed of quartz as the dominant mineral phase, calcite and siderite as the carbonate minerals, K-feldspar, smectite, and minor muscovite. The porosity of the sample is 24.84%. Table 1 contains details of the mineral composition.

**Table 1.** Properties of the Paluxy Formation Obtained from Multiscale Imaging of the Sample and Used in Prior Reactive Transport Simulations (Abundance, Volume Fraction, and Surface Area from Qin and Beckingham, 2019) and Rate Constants (Superscripts) for the Respective Mineral Phases as Obtained from the Literature

Mineral	Ideal chemical formula	Abundance (%)	Volume fraction	Surface area (m^2^g^−1^)	Log rate constant (mol s^−1^ m^−2^)
Quartz (Knauss and Wolery, 1988; Brady and Walther, 1990)	SiO_2_	76.45	0.5740	2.59E−2	−11.60
Calcite (Alkattan *et al.*, 1998)	CaCO_3_	9.63	0.0724	1.42E−3	−4.21
K-Feldspar (Bevan and Savage, 1989)	KAlSi_3_O_8_	3.50	0.0263	1.15E−3	−11.65
Smectite (Amram and Ganor, 2005)	(Na,Ca)_0.33_(Al,Mg)_2_(Si_4_O_10_)	8.23	0.0619	1.63E+1	−13.35
Muscovite (Oelkers *et al.*, 2008)	KAl_2_(Si_3_AlO_10_)(OH)_2_	0.31	0.0023	1.10E+0	−12.67
Siderite (Golubev *et al.*, 2009)	FeCO_3_	1.98	0.0141	6.49E−4	−5.69

### Reactive transport simulations

Coupling of solute transport, flow, and multiple species kinetic evolution for an injection-only and injection–extraction systems were simulated using CrunchFlow, a multicomponent reactive transport simulation code (Steefel *et al.*, 2015). In this study, a one-dimensional transient reactive transport model was developed focusing on the single-phase reactive zone contiguous to the two-phase CO_2_-brine zone in the storage aquifer (Fig. 1). Previous investigation of fluid-rock reactivity in the two-phase (Tutolo *et al.*, 2015) and single-phase (Huq *et al.*, 2015) flooding of acidified brine in core samples demonstrates that the single-phase region is anticipated to be the region with the most extensive geochemical reactions. As such, it was selected as the region of focus for this work.

A 47-cell model system is considered here to define the brine saturated region adjacent to the CO_2_ cushion gas bubble. The first cell, the one closest to the injection well, is a ‘ghost’ cell treated as a boundary condition where the formation brine equilibrates with CO_2_ using the improved CO_2_ solubility model in aqueous solution by Duan *et al.* that accounts for high pressure and temperature conditions (Duan *et al.*, 2006). The model assumes a constant partial pressure of CO_2_ in this cell. The 45 internal cells are defined as initially identical porous media cells with a total length of 15 cm. These cells are initialized according to the aquifer mineralogy, mineral surface areas, and porosity characterization results in Qin and Beckingham (2021). The last cell is another “ghost” cell, which is also treated as a boundary condition that serves as the influent fluid source during the extraction cycle.

**FIG. 2.**
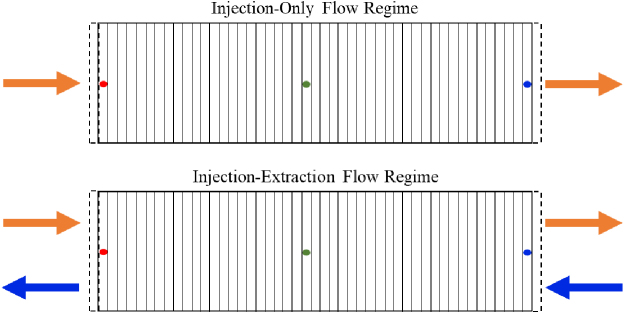
Schematic of the model setup for the injection-only flow regime and injection–extraction flow regime showing the direction of injection flow cycle (*orange arrow*) and extraction flow cycle (*blue arrow*). Also shown is the conceptualized location of the upstream (*red marker*), midstream (*green marker*), and downstream (*blue marker*) mineral cells, which are utilized for comparing reactive transport simulation results. The *dotted lines* illustrate the boundary conditions.

Simulations consider the flow of the acidified brine through the 45 mineral cells, tracking the concentration of major ion species, mineral volume fractions, and porosity at three mineral cell locations designated as upstream, midstream, and downstream mineral cells as shown in Fig. 2. The upstream location is in the first internal grid cell, the midstream is the central internal grid cell, while the downstream location is the furthest internal grid cell from the source of injection. Advective dominated flow through the mineral cells is simulated with flux continuity across the boundary using a constant flow rate of 0.489 m/day (Gelhar *et al.*, 1992). The brine flow rate was estimated based on extrapolation of modeling results from a field scale simulation considering brine velocities at the boundary of an injected CO_2_ plume in a sandstone formation (Zhang and DePaolo, 2017). Based on the geothermal gradient at Kemper, Mississippi and a typical pressure gradient, a reservoir temperature and pressure of 50°C and 100 bar are used for the simulation, respectively (Nathenson and Guffanti, 1988; Bachu, 2000; Reysa, 2015). The initial brine compositions of major primary species shown in Supplementary Table S1 were determined by simulating the equilibration of 1 M NaCl with quartz, calcite, K-feldspar, siderite, muscovite, and smectite (Table 1) for 10,000 years under reservoir temperature and pressure (Qin and Beckingham, 2021). The influent brine composition was then determined by equilibration with CO_2_ in the “ghost” cell. The CO_2_ concentration and initial pH of the brine after CO_2_ equilibration are 1.01 mol/kg and 3.17, respectively. The aqueous activity coefficients of the brine were obtained using the extended Debye-Huckel model.

Simulations consider injection-only and injection–extraction flow cycling for a 24-h cycle over a 4-month study period. The injection-only scenario reflects a CO_2_ storage system where CO_2_ is injected for a specified period of time and the injected CO_2_ remains indefinitely in the formation. The injection–extraction simulation is representative of the operational energy storage system where a cyclic flow pattern is used to cycle between energy storage (injection) and recovery (extraction). The injection-only simulation is modified from a study that investigates the influence of surface area on the rate of mineral reactivity during CO_2_ sequestration by Qin and Beckingham (2021). In these simulations, a CO_2_ acidified brine with constant composition, simulated using initial brine composition equilibrated with CO_2_ gas, flows away from the well. The Qin and Beckingham simulations consider geochemical evolution over 20 years in a 3 cm three-grid mineral cell where in this work 45 mineral cells are considered over 15 cm to investigate the geochemical reactions adjacent to the cushion gas bubble during the injection-only flow regime and injection–extraction flow regime.

The injection–extraction cycle starts with 12 h of injection flow away from the well followed by 12 h of extraction flow toward the well. This corresponds to a continuous operation system composed of a constant injection and extraction process corresponding to a CES system used daily for power generation (Fleming *et al.*, 2018). It should be noted that there are other CES operational models, including some that include shut-in periods between injection and extraction (Allen *et al.*, 1983; Pfeiffer *et al.*, 2017). During the first cycle, the influent is the initial brine composition equilibrated with CO_2_ as discussed above. The composition of the returning fluid, and subsequent influent for the remaining injection periods, is based on the effluent of the preceding flow regime as the brine is recycled through the system. For each injection period, the recycled influent is equilibrated with CO_2_ at the brine-cushion gas boundary.

Mineral reactions are simulated in CrunchFlow utilizing parallel rate laws to account for pH dependence and the effects of hydroxyl or electrolyte on the simulated reaction process (Steefel and Molins, 2016). The corresponding rate equation is given by:


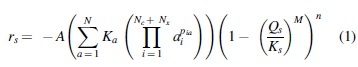


where *r_s_* is the reaction rate, *A* is the reactive surface area of a constituting mineral in the rock sample, *K_a_* is the equilibrium dissolution rate constant for the ‘*a'*th parallel reaction, *N* is number of parallel reactions, *p_ia_* is an exponent that gives the dependence of a species *i* on the ‘*a'*th parallel reaction, 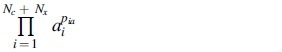 explains the degree of equilibrium effect of ions in solution, *n* and *M* are exponents which are experimentally determined to explain nonlinear dependence of the affinity term, *K_s_* is the equilibrium constant, and *Q_s_* is the ion activity product for the rock–water interaction. The rate constants which incorporate all geochemical dependencies relevant to the study were obtained from literature data and extrapolated to the reservoir temperature condition and reactive surface areas approximated as mineral accessible surface areas in Qin and Beckingham (2019). The pH of the system was determined using charge balance.

## Results and Discussion

The evolution of minerals is considered in simulations with an injection-only flow regime, corresponding to geologic CO_2_ sequestration, and an injection–extraction flow regime, corresponding to energy storage. In this study we present plots for 2 days and 4 months of operation in the Supplementary Data and paper body, respectively, that consider the temporal evolution of minerals, major ion concentrations, and porosity at three locations in the domain and the spatial evolution of minerals across the entire domain.

### Temporal mineral evolution

**FIG. 3.**
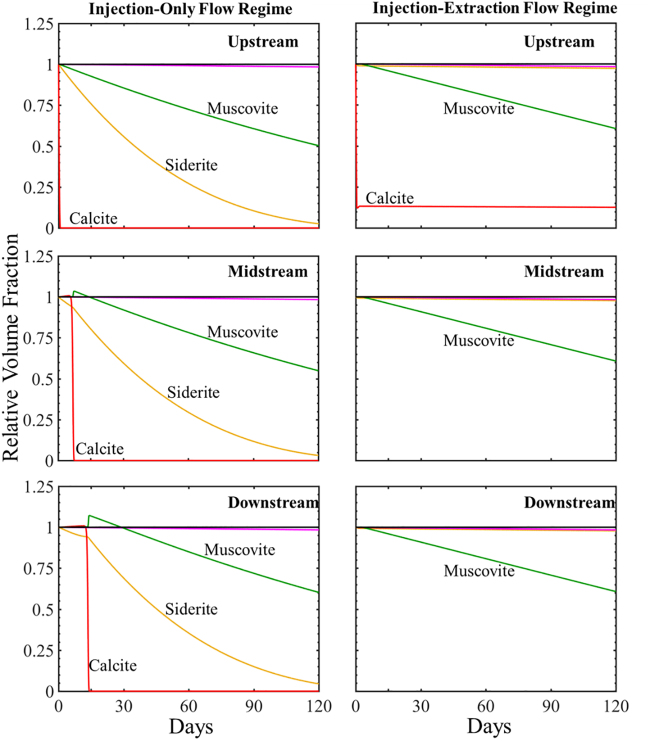
The simulated evolution of relative mineral volume fractions at three locations along the simulation domain over 120 days for the injection-only flow regime (*left*) and injection–extraction flow regime (*right*). Upstream is the location closest to the injection well and downstream is furthest (Fig. 2). The *red line* represents calcite, *yellow* siderite, *green* muscovite, *magenta* smectite, *black* quartz, and *blue* K-feldspar.

The simulated evolution of mineral volume fractions at three locations in the simulation domain is shown in the Supplementary Figs. S1 and S2 for individual minerals 2 and 120 days and in Fig. 2 for all minerals for 120 days (120 cycles). Mineral evolution is expressed in terms of mineral volume fraction (Supplementary Figs. S1 and S2) and relative volume fractions (Fig. 3) that correspond to the ratio of mineral volume fraction over initial mineral volume fraction for each phase. Values of relative volume fractions greater than one signify precipitation, and values less than one indicate dissolution.

#### Injection-only flow regime

As the CO_2_-saturated brine flows into the system, calcite, siderite, and smectite rapidly begin to dissolve as indicated by the decrease in relative volume fractions of each phase (seen more clearly in 0–0.5 day, Supplementary Fig. S1). After 0.5 day, muscovite begins to dissolve. Calcite and muscovite dissolution only occur in the upstream cell over the first 2 days, while siderite and smectite dissolve throughout the simulation domain where the dissolution rate of siderite is highest closest to the injection well (upstream location) which remains unbuffered. Quartz and K-feldspar remain stable throughout the simulation domain over the first 2 days.

The simulated evolution of formation minerals for over 120 days (Fig. 3) follows similar trends to that observed at short times. Calcite dissolution at the inlet initially results in slight calcite precipitation downstream that later dissolves. Complete dissolution of calcite occurs at 23, 181, and 258 h for the up-, mid-, and downstream mineral cells, respectively. Siderite continuously dissolves throughout the simulation domain. The average dissolution rate of siderite increases after complete dissolution of calcite in the system and decreases as siderite nears depletion. At early times, muscovite precipitates then begin dissolving following complete dissolution of calcite in each cell. Muscovite precipitation, however, does not occur in the upstream mineral cell. SiO_2_ is predicted to precipitate, while K-feldspar remains stable. Precipitation of additional secondary mineral phases was also investigated. Conditions were observed favorable for possible precipitation of albite, chalcedony, chlorite, and kaolinite (Supplementary Fig. S3) although to very small volume fractions (<3 orders of magnitude of primary minerals). Throughout the simulation duration, conditions continuously favor chalcedony and chlorite precipitation, indicated by saturation indices greater than one. Conditions favor albite precipitation at early times and dissolution as time progresses. Kaolinite initially precipitates throughout the domain and dissolves closer to the injection well as time progresses.

#### Injection–extraction flow regime

The simulated evolution of formation minerals for the injection–extraction flow regime corresponding to energy storage is shown in Supplementary Figs. S1 and S2 for durations of 2 and 120 days. The mineral response is the same during the first 12 h as the injection-only system. After 12 h, the system evolves discretely differently as the cyclic flow pattern begins. During the extraction flow regime, brine recycles through the system. In the first extraction flow regime, the higher resolution plots of Supplementary Fig. S1 show that between 0.5 and 1 day, there is little change in calcite mineral volume fraction as the brine is almost in equilibrium with respect to calcite. Siderite and smectite continue to dissolve, while muscovite begins to dissolve in the upstream location and no changes in quartz and K-feldspar volume fractions occur. The dissolution rate of siderite is greatest in the cell furthest from the injection well and decreases in the cells closer to the injection well. After 1 day, the second 12-h injection cycle begins corresponding to brine recycling with replenished acidity as influent brine is saturated with CO_2_. This results in continued dissolution of siderite and smectite, but does not result in increased dissolution rates as the ion concentrations in the recycled brine limit reactions. In comparison with the injection-only scenario, initial dissolution rates (Supplementary Fig. S1) in the injection–extraction flow regime are smaller and ultimately reduce the extent of dissolution. The dissolution rate for smectite, however, is the same for both flow conditions.

As time progresses, calcite and smectite dissolve continuously throughout the simulation, although at slow rates at longer times (higher resolution plots shown in Supplementary Fig. S2). Smectite is also dissolving with a rate dissolution similar to that in the injection-only flow conditions (Supplementary Fig. S2). As in the injection-only simulation, SiO_2_ slowly precipitates and K-feldspar remains stable. The extent of quartz precipitation for the injection–extraction flow regime, however, is slightly less than for the injection-only flow regime (Supplementary Fig. S2). Potential additional secondary mineral phases include albite, chalcedony, chlorite, and kaolinite (Supplementary Fig. S4). As indicated by saturation indices, conditions favor precipitation throughout the simulation domain, although to small volume fractions. The volume fraction of most dominant precipitate is also more than three orders of magnitude less than the primary minerals. In this study, continuous precipitation is favored, which is distinctly different compared with the injection-only flow conditions where only chlorite and chalcedony were stable throughout the simulation time and domain.

### Spatial mineral evolution

The evolution of the mineral volume fractions across the domain length for the two flow regimes is discussed below with respect to the number of pore volumes (PVs) of fluid that have passed through the domain.

#### Injection-only flow regime

**FIG. 4.**
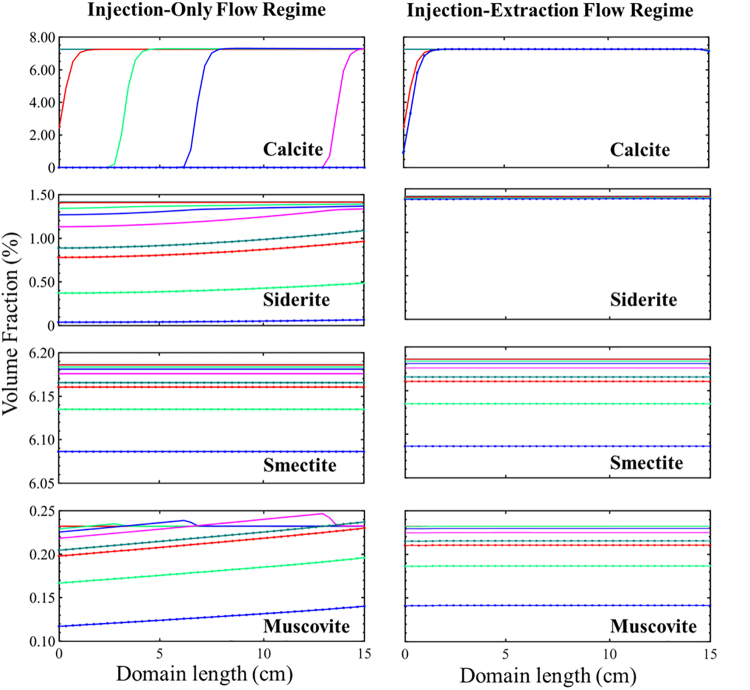
The simulated evolution of mineral volume fractions with increasing number of PV of CO_2_ acidified brine flowing through the simulation domain over 120 days for the injection-only flow regime (*left*) and injection–extraction flow regime (*right*). Zero PV is the initial condition and 391 PV is the last PV to flow through the porous media. *Dark green* represents 0 PV, *red* 1 PV, *light green* 10 PV, *blue* 20 PV, *magenta* 40 PV, *dotted dark green* 80 PV, *dotted red* 1 PV, *dotted light green* 200 PV, and *dotted blue* 391 PV. PVs, pore volumes.

The simulated spatial evolution of formation minerals with respect to PVs for the injection-only flow regime is given in Fig. 4 and shows nonuniform dissolution of calcite and siderite. Variations in calcite dissolution show that calcite successively dissolves, and is consumed, from the inlet to the outlet. Siderite dissolution is initially larger near the injection well and becomes more uniform throughout the simulation domain as simulations progress. This nonuniform dissolution pattern of calcite and siderite mineral is expected as dissolution of these minerals buffers the acidity, creating conditions where calcite and siderite are more stable. The high reaction rate of calcite results in rapid depletion of calcite after contact with acidified brine that prevents downstream calcite dissolution until it is completely consumed upstream. This results in nonuniform calcite volume fractions across the domain until all calcite are consumed, after more than 40 PVs. In comparison, the lower dissolution rate of siderite results in siderite dissolution throughout the simulation domain earlier in the simulation. Unlike calcite, siderite dissolution approaches uniform extents across the domain length as simulations progress.

Large variations in muscovite across the simulation domain can also be observed. Initially, muscovite precipitates, coupled with calcite dissolution. As calcite is depleted, muscovite then precipitates. Once calcite is consumed, muscovite dissolves throughout the simulation domain to varying extents with the largest reduction in muscovite volume fraction near the injection well.

Smectite, K-feldspar, and quartz do not vary across the simulation domain. Smectite dissolves continuously throughout the simulation domain as a result of smectite's lower dissolution rate in comparison to calcite and siderite; Quartz precipitates uniformly throughout the simulation domain throughout the duration of the simulations. K-feldspar is constant throughout the simulation. The spatial mineral evolution of quartz and K-feldspar is shown in Supplementary Figure S3.

#### Injection–extraction flow regime

The simulated spatial variation of mineral volume fractions in the injection–extraction flow regime is much less than in the injection-only flow regime, as shown in Fig. 4. In this study, the recycling process significantly reduces the rate and extent at which calcite and siderite dissolve. Some spatial variation is evident for calcite with increased dissolution near the injection well and no dissolution of calcite further in the simulation domain. While CO_2_ saturated brine enters the system during each injection half-cycle, the elevated ion concentrations from earlier calcite dissolution limit additional dissolution. Siderite dissolves uniformly in the domain throughout the simulation but to a much lower extent than in the injection-only flow regime. Smectite also uniformly dissolves, facilitated by its slower dissolution rate and the continuous acidic conditions. The rate of smectite dissolution is the same in the injection-only and injection–extraction flow conditions. K-Feldspar is stable throughout the domain and simulation like the injection-only flow regime. Muscovite dissolves uniformly during cyclic flow conditions with no initial precipitation because of the absence of rapid calcite dissolution. Overall, the extent of muscovite dissolution is less than in the injection-only flow regime, particularly closer to the injection well. Quartz uniformly precipitates throughout the domain to a slightly lesser extent than the injection-only flow regime, a consequence of overall reduced muscovite dissolution.

### Evolution of major ion concentrations

#### Injection-only flow regime

**FIG. 5.**
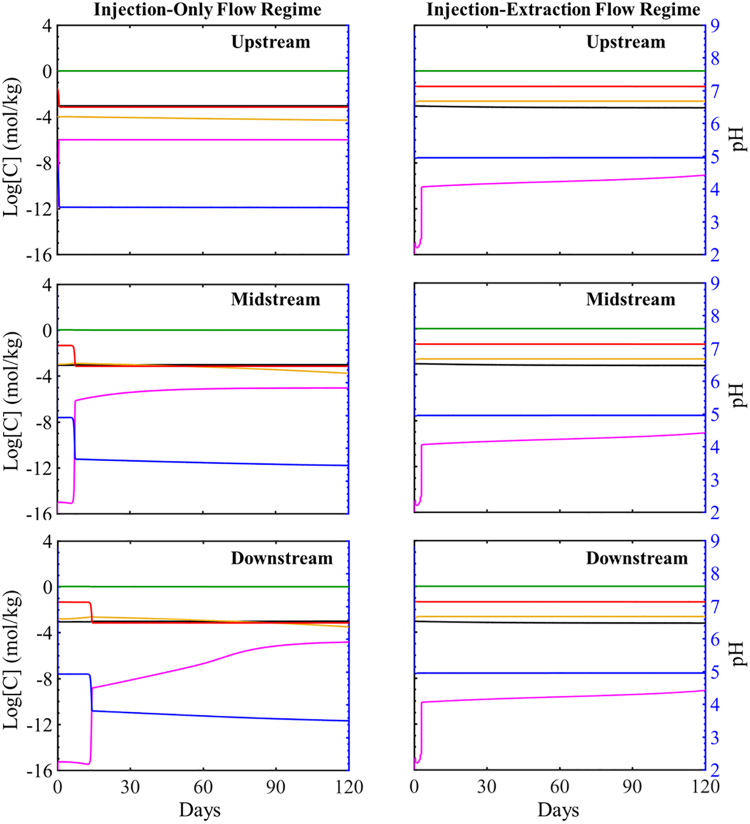
Simulated evolution of major ion concentrations and pH over the 120-day study period for the injection-only flow regime (*left*) and injection–extraction flow regime (*right*). Upstream is the cell closest to the injection well, and downstream is the furthest. The *red* represents calcium, *yellow* iron, *green* total CO_2_, *magenta* magnesium, *black* silica, and *blue* pH.

The simulated evolution of individual major ion concentrations in the brine during the injection-only flow regime is shown in Supplementary Figure S5 for two days and Supplementary Figure S6 for 120 days and Figure 5 show the comparative concentration of major ions for the 120-day simulation duration. At the start of the simulation, the introduction of acidified brine results in a sharp increase in calcium, iron, and magnesium and a reduction in pH (Supplementary Fig. S5). The increase in calcium is from the rapid dissolution of calcite, which results in an increase of calcium concentration of two orders of magnitude relative to its initial concentration. Calcite dissolution concurrently buffers the pH and results in an increase in pH of the brine from 3.42 (pH of entering brine) to 4.8, 4.9, and 4.9 in the upstream, midstream, and downstream locations, respectively. After the initial rapid change, the calcium ion concentration in the upstream location starts to decrease due to the reduction in calcite dissolution with decreasing calcite volume fraction in these cells. This is closely coupled with pH where the pH gradually drops as calcite is depleted and the extent of buffering is reduced.

As calcite is depleted, calcium concentrations return to background levels, first in the upstream cell and later in the midstream and downstream locations. The increase in iron and magnesium concentrations reflects dissolution of siderite and smectite where concentrations are lowest in the grid cell closet to the injection well and increase with distance from the well. As siderite is depleted after tens of days, iron concentrations decrease. Magnesium remains at a constant elevated concentration in the upstream location, while mid- and downstream concentrations continue to increase, reflecting the constant dissolution of smectite and eventually muscovite following initial muscovite precipitation. No change in aqueous silica or potassium concentrations occurs within the first 2 days.

#### Injection–extraction flow regime

The species evolution for the injection–extraction flow regime initially evolves similar to the injection-only flow regime where increases in iron, calcium, magnesium, and silica are observed as calcite, siderite, and smectite dissolve. After 0.5 day, simulations begin to diverge as the near-saturated brine is recycled as the flow reverses and the first extraction cycle begins (Supplementary Fig. S5). The returning brine contains relatively high concentrations of the ions from the minerals that dissolved during the first injection half-cycle. As such, the returning concentrations reflect effluent ion concentrations in the downstream grid cell at the end of the previous injection period. The high concentration of calcium in recycled brine prevents further calcite dissolution. Iron and magnesium concentrations fluctuate but maintain undersaturated conditions with respect to siderite and smectite dissolution, facilitating additional dissolution. No change in SiO_2_ concentration occurs. At 1 day, the second injection cycle begins where CO_2_ concentrations are refreshed in the solution, as during each injection cycle. This replenished acidity results in additional increases in iron concentration from more siderite dissolution and further buffering of the system pH, while calcium ion concentrations remain stable. SiO_2_ concentrations continue to be elevated over the first 2 days.

Over longer times, calcium concentrations remain constant at elevated levels (Fig. 5) in each grid cell. Magnesium concentrations continuously increase throughout the simulation domain reflecting continued smectite dissolution throughout the study period. The initially oversaturated silicate concentration begins to decrease after ∼30 days as quartz begins to precipitate, although the variations are overall small. Iron concentrations gradually increase as siderite continuously dissolves.

### Porosity evolution

**FIG. 6.**
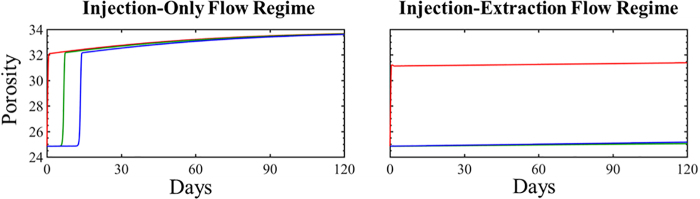
The simulated evolution of mineral porosity of the core sample in three different grid cells over the 4-month study period for the injection only flow regime and injection–extraction flow regime. The upstream location is closest to the injection well, and downstream is furthest. The *red* represents the upstream location, *green* the midstream location, and *blue* the downstream location.

The simulated evolution of porosity for the injection–extraction flow regime and injection-only flow regimes is shown in Fig. 6. The porosity evolution serves to quantify the total effect of the mineral dissolution and precipitation reactions occurring in the sample following injection of CO_2_.

In the injection-only simulations, porosity rapidly increases throughout the simulation domain within the first 15 days (Supplementary Fig. S7) of CO_2_-acidified brine injection and continuously increases throughout the study period. This is largely a result of dissolution of calcite and siderite. At the end of the study period, the porosity has increased to 33.6%, from 24.8%, throughout the simulation domain. During the injection–extraction flow regime for the cyclic flow conditions, dissolution of calcite, siderite, smectite, and muscovite results in an overall increase in porosity. The porosity increase is highest near the injection well and decreases away from the well, largely due to spatial variations in calcite dissolution where 87.4%, 1.6%, and 0.1% of calcite dissolve in the upstream, midstream, and downstream locations at the end of the 120 cycles. This results in final porosities of 31.4%, 25.1%, and 25.2% for the three locations at the end of the 4-month study period.

After the initial cycle, there is little additional variation in porosity as little additional dissolution occurs. The overall porosity increase in the cyclic flow conditions is small in comparison with the injection-only flow conditions, with the exception of the location closest to the cushion gas boundary. This is because brine recycling maintains elevated ion concentrations and limits mineral dissolution as injection–extraction cycles progress, even under CO_2_-saturated conditions. Both simulation systems result in rapid large increases in porosity near the injection well. However, it should be noted that this may be dependent on the model domain where reservoir scale simulations have observed much smaller variations in porosity near the injection well due to simulated near-well pH buffering in the larger domains (Zhang and DePaolo, 2017; Wang *et al.*, 2019).

## System Implications

Geochemical reactions are anticipated in porous aquifers utilized for developing subsurface technologies, such as CO_2_ sequestration and subsurface energy storage. The potential rate and extent of these reactions in subsurface energy storage systems and the resulting implications on operational performance, however, have largely not been investigated while numerous works have considered reactions in the context of CO_2_ sequestration. Energy storage in porous saline aquifers and geologic CO_2_ sequestration systems have many system similarities, including target reservoir formations. However, there is a major difference in the operational flow regime of energy storage systems that may impact the gas dissolution zone initiated during the lifecycle of the project (Allen, 1981; McGrail *et al.*, 2011). In this study, reactive transport simulations are developed and leveraged to compare the reaction pathways during CO_2_ sequestration and subsurface energy storage to predict the difference in potential geochemical reactions and implications for operational efficiency.

Geochemical reactions play an important role in these subsurface energy systems, impacting potential associated environmental risks and the operational efficiency of the system. In terms of risk, previous investigations of CO_2_ sequestration systems have highlighted the need to evaluate the risk of leakage and land subsidence, two adverse effects that are largely controlled by geochemical reactions. The formation of leakage pathways in caprock formations (Fitts and Peters, 2013) following CO_2_ injection can result in flow of brine or injected fluids to overlying formations and endanger natural resources and protected entities, including drinking water aquifers (Bauer *et al.*, 2013). Land subsidence may jeopardize the integrity of the site of operation and has been observed in pilot CO_2_ systems (Onuma and Ohkawa, 2009). In terms of operational efficiency, geochemical reactions may also alter the porosity and permeability of the formation and, thus, the injectivity during the operational life of the energy storage system. In general, dissolution at the plume boundary would increase the storage volume and injectivity but may have adverse effects in terms of migration of the cushion or storage gas further into the formation and a corresponding reduction in pressure and energy recovery. Precipitation at the boundary may limit injectivity but can also serve to limit migration of the plume into the formation and increase efficiency of energy recovery by maintaining pressurization.

The results of this study show that geochemical reactions will occur in energy storage systems when CO_2_ is utilized as a cushion gas. Both mineral dissolution and precipitation reactions are anticipated in the single-phase brine-saturated region adjacent to the cushion gas plume. The dissolution potential for the case of CO_2_ sequestration, however, supersedes that of the cyclic flow regime of the CES system. The cyclic flow pattern of energy storage and recovery results in a high concentration of dissolved ion concentrations as CO_2_ saturated brine flows away from and toward the well, reducing the extent of dissolution at the plume boundary in comparison to that occurring in CO_2_ sequestration conditions. Similar observations with regards to limited dissolution have been observed experimentally in studies with low water–rock ratios where highly dissolved ion concentrations limited the extent of dissolution (Huang *et al.*, 1986).

The reduced dissolution extent in the cyclic flow conditions limits porosity variation as reactions predominantly occur during the initial cycle and only impact carbonate minerals after the first injection cycle. This means that the storage volume and injectivity will largely remain constant after the first cycle. As properties are anticipated to be less dynamic, this can potentially reduce the risk and likelihood of overpressurization of the aquifer during the life cycle of operation by improving the predictability of the system. If conditions continuously favored dissolution, as in the CO_2_ sequestration scenario, this would result in a constant increase of porosity and allow migration of the working and/or cushion gas plume further into the formation. This would result in a fluctuation of the pressure of the system as the injected fluid migrates further into the reservoir. This condition would require more frequent monitoring during operation and more frequent injections of additional cushion gas to ensure sufficient pressure for energy recovery. However, the increased dissolution may additionally allow for the mineralization of the injected CO_2_, which is a means of secure CO_2_ sequestration. This secondary mineral precipitation may decrease porosity and permeability.

While limited reaction rates and extents were observed in the cyclic flow simulations here over the 4-month study period, the difficulty in accurately simulating reaction rates and extents in field scale systems should be noted. In part, this is due to uncertainties in the parameters used for modeling (Black *et al.*, 2015; Bourg *et al.*, 2015; Zhang *et al.*, 2020), namely accurate estimation of reaction rate constants and mineral surface areas. Rate constants vary widely with pH, as much as 8 orders of magnitude between pH 3 and 8 (Black *et al.*, 2015; Zhang and DePaolo, 2017). However, rate constants are anticipated to vary by only approximately one order of magnitude for the simulated pH values here following CO_2_ injection, pH approximately 3–5 (Zhang and DePaolo, 2017).

Estimates of mineral reactive surface areas depend largely on approximation approach where variations in estimation method yield as much as seven orders of magnitude variation in surface area values (Black *et al.*, 2015; Bourg *et al.*, 2015; Beckingham *et al.*, 2017). For the study period considered here, variations in surface area may result in differences in reaction rates and slight differences in porosity, as determined in sensitivity simulations considering the impact of surface area approximation on the rate and extent of reactions for the geologic storage condition in this formation in Qin and Beckingham (2021). However, recent work considering reaction rates in porous media found that image obtained accessible surface areas best reflected the surface area of reacting mineral phases, and reaction rates were overestimated using other common approaches (Beckingham *et al.*, 2017). As such, mineral accessible surface areas determined using the same multiscale imaging approach (Qin and Beckingham, 2019) are used in the simulations here and are anticipated to reflect reaction rates and extents in porous media.

The impact of mineral dissolution and precipitation reactions on the operation lifecycle of these systems will largely depend on the corresponding evolution of permeability in the formation. While the evolution of porosity can be estimated based on changes in mineral volume fractions with the micro-continuum reactive transport simulations here, changes in permeability depend on location of mineral reactions within individual pores and the larger pore network. This, however, is not well understood. Based on the simulated permeability evolution, previous pore network modeling work has shown that permeability will likely range between 1000 and 2200 mD, in comparison to the initial permeability of 1555.4 mD, but may be more extreme depending on the spatial distribution of mineral reactions (Bensinger and Beckingham, 2020).

In the 4-month study period considered here, a significant difference in the simulated geochemical reactions and porosity evolution for the case of CO_2_ sequestration and CES using CO_2_ as the cushion gas is anticipated. It should be noted that this result is for an operational system composed of constant injection and extraction for 12 h each. In terms of operational cycles, injection–extraction periods can vary from hours to months and may include long storage periods (Allen *et al.*, 1983). In comparison to geologic CO_2_ sequestration, the extent, rate, and impact of geochemical reactions are limited in the single-phase zone of energy storage systems utilizing CO_2_ as a cushion gas. In the CO_2_ sequestration system, reactions progress continuously as undersaturated acidic formation brine flows through the aquifer and porosity increases continuously. The cyclic flow conditions of energy storage systems limit reactions such that a stabilized working system can be attained after only one cycle, making utilization of reactive cushion gases, including CO_2_, a viable alternative.

## Supplementary Material

Supplemental data

Supplemental data

Supplemental data

Supplemental data

Supplemental data

Supplemental data

Supplemental data

Supplemental data

Supplemental data
