# Supplementary material for: Assessment of Geochemical Limitations to Utilizing CO2 as a Cushion Gas in Compressed Energy Storage Systems
Source: Environ Eng Sci. 2021 Mar 17;38(3):115–26. doi: 10.1089/ees.2020.0345 (PMC7994420; doi:10.1089/ees.2020.0345)
Supplement: Supplemental data [file Supp_TableS1.docx]

**Table S1:** Simulated initial brine composition of the Paluxy formation.

| Primary species | Concentration (Mol/kg_w_) |
| --- | --- |
| HCO_3_^-^ | 7.53E-04 |
| SiO_2_(aq) | 8.87E-04 |
| Al^+++^ | 1.09E-06 |
| Fe^++^ | 4.56E-05 |
| Ca^++^ | 7.07E-04 |
| Mg^++^ | 6.19E-07 |
| K^+^ | 1.07E-04 |
| Na^+^ | 1.00E+00 |
| Cl^-^ | 1.00E+00 |
| SO_4_^--^ | 1.08E-4 |

The pH of the system was calculated by using charge balance. The initial pH is 8.82.
