# Supplementary material for: Assessment of Geochemical Limitations to Utilizing CO2 as a Cushion Gas in Compressed Energy Storage Systems
Source: Environ Eng Sci. 2021 Mar 17;38(3):115–26. doi: 10.1089/ees.2020.0345 (PMC7994420; doi:10.1089/ees.2020.0345)
Supplement: Supplemental data [file Supp_Data.docx]

Supporting Information:

**Assessment of Geochemical Limitations to Utilizing CO_2_ as Cushion Gas in Compressed Energy Storage Systems**

Chidera O. Iloejesi; Lauren E. Beckingham^*^

Department of Civil Engineering, Auburn University, Auburn, Alabama, 36849.

***Corresponding Author Tel:** 334-844-6260; **e-mail:** leb0071@auburn.edu

**List of Figures**

The simulated evolution of mineral volume fraction in three different grid cells over the first two cycles for the injection-only flow regimes and injection-extraction flow regimes. Upstream is closest to the source of CO_2_ injection and downstream is furthest……………………………………………………………………………………………S1

The simulated evolution of mineral volume fraction in three different grid cells over the 4 months study period for the injection-only flow regime and injection-extraction flow regime. Upstream is closest to the source of CO_2_ injection and Downstream is furthest…………………………………………………………………………………………...S2

The simulated evolution of mineral volume fraction with increasing number of pore volumes (PV) of CO_2_ acidified brine flowing through the core sample over 120 days for the injection-only flow regime (left) and injection-extraction flow regime (right). 0 PV is the initial condition and 391 PV is the last pore volume to flow through the porous media. The color code for the minerals are dark green represent 0 PV, red represent 1PV, light green represent 10PV, blue represent 20PV, purple represent 40PV, dotted dark green represent 80 PV, dotted red represent 1PV, dotted light green represent 200PV, dotted blue represent 391PV……….. ………………………………………..………Figure S3

The simulated evolution of saturation index of the potential mineral precipitates in three different grid cells over the 4 months study period for the injection-only flow regime and injection-extraction flow regime. Upstream is closest to the source of CO_2_ injection and downstream is furthest……………………………………………………………….S4

The simulated evolution of major ion concentrations and pH of the porous formation in three different grid cells over the first two cycles for the injection-only flow regimes and injection-extraction flow regimes. Upstream is closest to the source of CO_2_ injection and downstream is furthest……………………………………………………………….S5

The simulated evolution of major ion concentrations and pH in three different grid cells over the 4 months study period for the injection-only flow regime and injection-extraction flow regime. Upstream is closest to the source of CO_2_ injection and downstream is furthest………………………………………………………………………………….S6

The simulated evolution of mineral porosity of the porous formation in three different grid cells over the first fifteen days for the injection-only flow regimes and injection-extraction flow regimes. Upstream is closest to the injection well and downstream is furthest………………………………………………………………………………………S7
