# Supplementary material for: Assessment of Geochemical Limitations to Utilizing CO2 as a Cushion Gas in Compressed Energy Storage Systems
Source: Environ Eng Sci. 2021 Mar 17;38(3):115–26. doi: 10.1089/ees.2020.0345 (PMC7994420; doi:10.1089/ees.2020.0345)
Supplement: Supplemental data [file Supp_FigS2.docx]

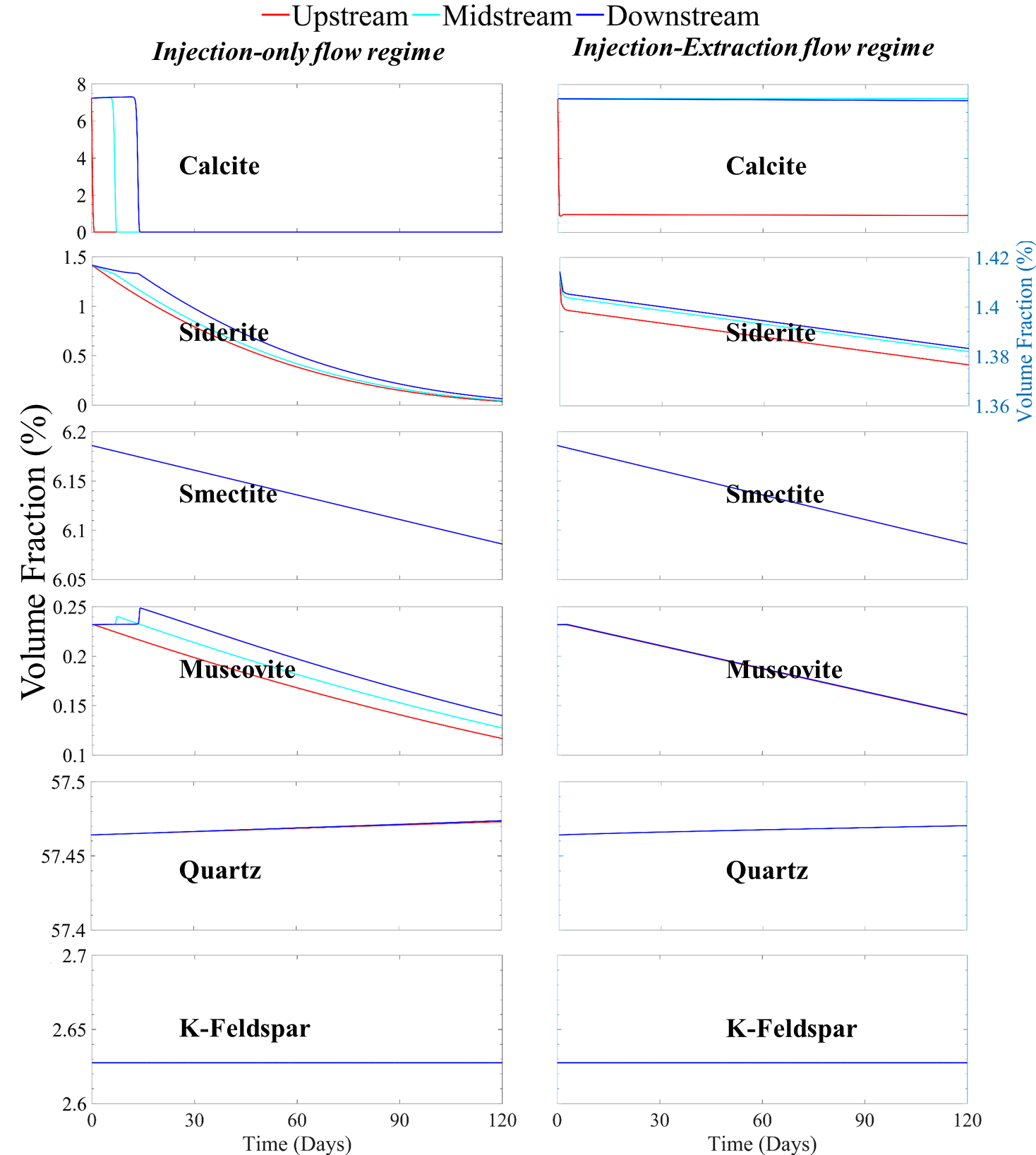


Figure S2: The simulated evolution of mineral volume fraction in three different grid cells over the 4 months study period for the injection-only flow regime and injection-extraction flow regime. Upstream is closest to the source of CO_2_ injection and downstream is furthest.
