# Supplementary material for: Assessment of Geochemical Limitations to Utilizing CO2 as a Cushion Gas in Compressed Energy Storage Systems
Source: Environ Eng Sci. 2021 Mar 17;38(3):115–26. doi: 10.1089/ees.2020.0345 (PMC7994420; doi:10.1089/ees.2020.0345)
Supplement: Supplemental data [file Supp_FigS3.docx]

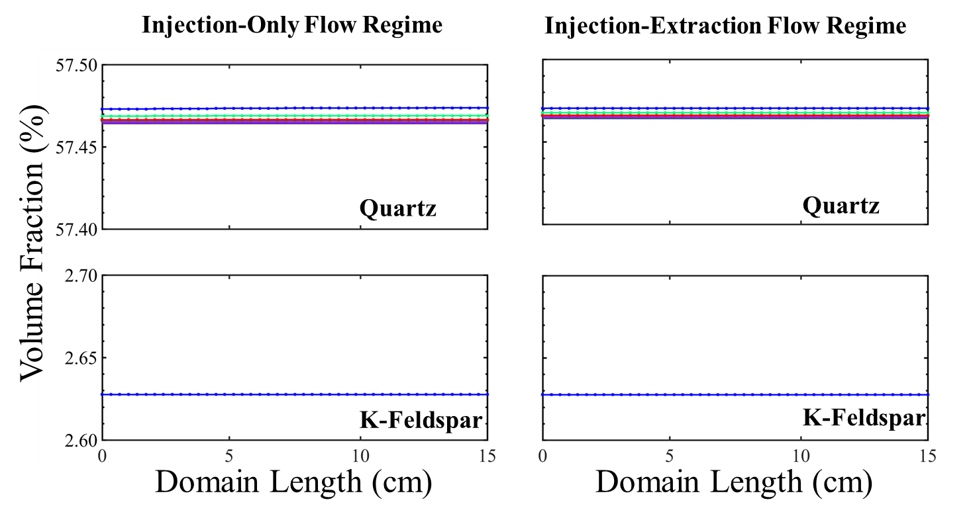


Figure S3: The simulated evolution of mineral volume fractions with increasing number of pore volumes (PV) of CO_2_ acidified brine flowing through the simulation domain over 120 days for the injection-only flow regime (left) and injection-extraction flow regime (right). 0 PV is the initial condition and 391 PV is the last pore volume to flow through the porous media. Dark green represents 0 PV, red 1PV, light green 10PV, blue 20PV, magenta 40PV, dotted dark green 80 PV, dotted red 1PV, dotted light green 200PV, and dotted blue 391PV.
